# The tmRNA website

**DOI:** 10.1093/nar/gku1109

**Published:** 2014-11-05

**Authors:** Corey M. Hudson, Kelly P. Williams

**Affiliations:** Sandia National Laboratories, Department of Systems Biology, Livermore, CA 94551, USA

## Abstract

The transfer-messenger RNA (tmRNA) and its partner protein SmpB act together in resolving problems arising when translating bacterial ribosomes reach the end of mRNA with no stop codon. Their genes have been found in nearly all bacterial genomes and in some organelles. The tmRNA Website serves tmRNA sequences, alignments and feature annotations, and has recently moved to http://bioinformatics.sandia.gov/tmrna/. New features include software used to find the sequences, an update raising the number of unique tmRNA sequences from 492 to 1716, and a database of SmpB sequences which are served along with the tmRNA sequence from the same organism.

## INTRODUCTION

tmRNA uses both tRNA-like and mRNA-like properties during the process of *trans*-translation (1). When a ribosome stalls on a non-stop mRNA, alanine-charged tmRNA enters as a substrate for peptidyl transfer. The ribosome switches from the defective mRNA to the ‘resume codon’ of tmRNA and continues translation, adding a peptide tag to the protein product that is a signal for proteolysis. This frees the stalled ribosome and marks the non-stop mRNA for degradation. The protein SmpB is a partner throughout this process (2), bound to tmRNA and occupying space normally occupied by the anticodon stem-loop (3,4). tmRNA genes have been found in nearly all bacteria (except for six recalcitrant genomes) and some organelles; *smpB* genes have been found in all bacterial genomes studied to date (although sometimes with severe defects) and in eukaryotic nuclear genomes with signals targeting transport into organelles that encode tmRNA (5). As an aid to research on *trans*-translation, we present The tmRNA Website, a repository of tmRNA and SmpB sequences and related information.

## THE tmRNA WEBSITE DESCRIPTION

The tmRNA Website provides several research tools resource for investigating tmRNA and their associated *smpB*s. tmRNA sequences were discovered using a combination of existing tools (tRNAscan-SE (6), BRUCE (7) and ARAGORN (7)) as well as the program rFind.pl, which uses our full- and terminus-sequence tmRNA databases with BLASTN to find additional two-piece tmRNAs and accurately determine their termini. These four primary programs are wrapped with post-processing by tFind.pl, which is available on the software page of the tmRNA Website (http://bioinformatics.sandia.gov/tmrna/software.html). This pipeline was applied to complete genomic sequences for 2168 organisms, 1755 additional plasmids and 581 additional viruses (137, 44 and 38 of which respectively were archaeal, the rest bacterial), all downloaded from RefSeq (8) in November 2012. The products were inspected and merged with the previous database contents. The current database contains 1716 unique tmRNA sequences (1454 are one-piece tmRNA and 262 are two-piece tmRNA (9)); these encode 734 unique proteolysis tag sequences. The phylogenetic breakdown of unique sequences is 1594 bacterial, zero archaeal and 122 organellar tmRNA sequences (79 in oomycete and jakoid mitochondria, 42 in algal plastids and one in a chromatophore). Each of these sequences was used as a query in BLASTN searches against NCBI est, gss, htgs, nt, other_genomic, patnt, refseq_genome, tsa_nt and wgs databases (10), resulting in 9387 instances of perfect (although potentially incomplete) matches. These sequences were provided to RNAcentral (11) and as third-party annotation to the International Nucleotide Sequence Database Archive (Genbank/ENA/DDBJ) (12).

The tmRNA Website also provides SmpB amino acid sequences, each linked with its associated tmRNA. SmpBs were found using HMMER against the SmpB HMM from Pfam (13), and RPS-TBLASTN against five SmpB profiles (TIGR00086, cd09294, PRK0544, COG0691 and pfam01668) from Conserved Domain Database (14). The default threshold was used for the SmpB HMM. The thresholds for RPS-TBLASTN were set 1.4-fold above the highest score for a non-SmpB. Each case where a bacterial genome yielded no *smpB* was examined manually applying TBLASTN searches and manual search in the vicinity of the *ssrA* gene. One particularly recalcitrant case (*Hodgkinia cicadicola* TETUND1) was examined comparatively; *smpB* could be found in a newer genome of the same genus, allowing its identification throughout the genus (5).

tmRNA features are presented, including their length, sequence, location in the genome, their proteolysis tag and CCA coordinates and sequence, introns when present, and special aspects of two-piece tmRNAs. Instances of the same tmRNA in different genomes are noted. Where available, the sequences include images of secondary structures. SmpBs annotation includes the amino acid sequence, coordinates in the genome, and orientation and location relative to the *ssrA* gene.

Additionally, sequence alignments are presented for 632 tmRNA and 2258 distinct SmpBs; BLAST search tools are provided for tmRNA and SmpBs. The tmRNA identification software used here is freely available for public download and use.

**Figure 1.**
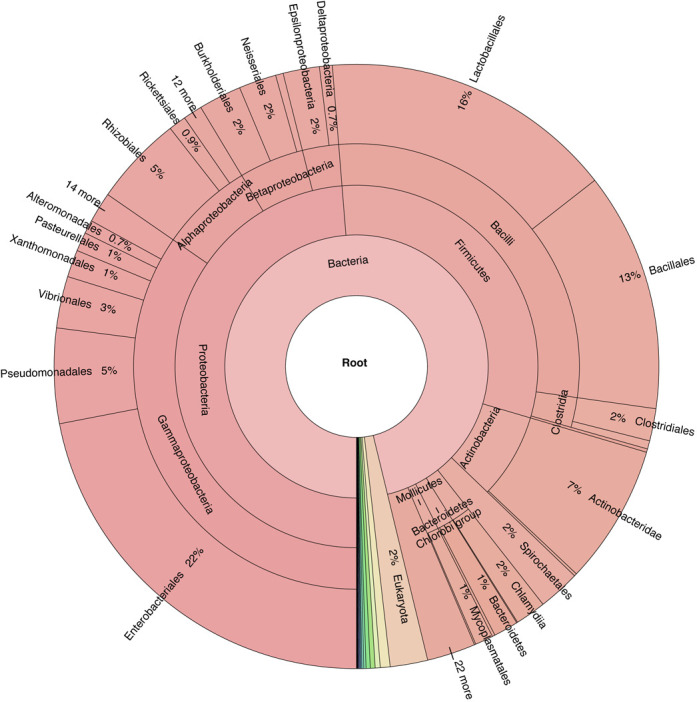
Taxonomic distribution of tmRNAs in the website. The Krona metagenome visualizer (15) is used to display 9387 tmRNA instances. In this visualization, higher taxonomic ranks are toward the center. In its dynamic form at our website, users click to restrict to lower ranks but with fuller presentation. It has been modified to also serve as a navigation tool for the website.

We have modified a dynamic metagenome taxonomy viewer, Krona (15), to enable navigation to individual tmRNA pages, while also providing a visual depiction of the phylogenetic distribution of tmRNA instances in the database (Figure 1).

## RELATED RESOURCES

Additional excellent online sources of tmRNA information are tmRDB (16), Rfam (17) and the RNAcentral consortium (11) to which we contribute. Our tmRNA annotations are also available as third-party annotation at the International Nucleotide Sequence Database Collaboration archives (GenBank/ENA/DDBJ).

## WEBSITE UPDATE

This major update of The tmRNA Website has greatly increased the number of unique tmRNA sequences relative to the previous version (18), from 492 to 1716. These were found in 9387 instances among public databases. The tmRNA Website annotates several key tmRNA features. It newly includes SmpB sequences and links them to their tmRNA partners, with 2258 unique sequences occurring in 4125 instances, including 24 potentially pseudogenized/frameshifted/truncated sequences. Also included is the software used here, containing the tools tFind.pl and rFind.pl.
